# New reduction technique simplified for surgical stabilisation of isolated manubriosternal dislocation: A case report and literature review

**DOI:** 10.7196/AJTCCM.2020.v26i4.022

**Published:** 2020-12-01

**Authors:** N N M Razafimanjato, E Rabezanahary, N Ratsimarisolo, M Ravoatrarilandy, T A Rajaonera, A Ahmad, H J L Rakotovao

**Affiliations:** 1 Thoracic Surgery Unit, Joseph Ravoahangy Andrianavalona Hospital, Antananarivo, Madagascar; 2 Imaging Medical Center, Joseph Ravoahangy Andrianavalona Hospital, Antananarivo, Madagascar; 3 Resuscitation Care Unit, Joseph Ravoahangy Andrianavalona Hospital, Antananarivo, Madagascar

**Keywords:** Biomechanics, Chest trauma, Manubrio-sternal dislocation, Median sternotomy, Sternum

## Abstract

Manubriosternal joint (MSJ) dislocation is often poorly tolerated, with pain, and both static and dynamic dysfunction in breathing. This injury is rare, and treatment includes both surgical and non-surgical interventions. Moreover, the treatment needs to be personalised to a specific case. We present a case of a 19-year-old Comorian man who had chest pains that were exacerbated by movements after falling from a tree. Careful physical examination revealed that the man had a ‘stair step’-looking deformity located at the anterior chest wall at the level of the MSJ. A computed tomography scan confirmed the diagnosis of manubriosternal disruption. The patient underwent a surgical intervention under general anaesthesia and had an uneventful recovery.

## Background

An unusual entity of dislocation of the manubriosternal joint (MSJ) represents 1.3 - 3.0% of all traumatic injuries.^[1]^ In the literature, there has been a lengthy discussion about non-operative and surgical approach for MSJ dislocation due to the paucity of data on the incidence of cases and no consensus was found. Based on a retrospective analysis of 1 124 patients involved in motor vehicle crashes over a period of 3 years, we noticed that the incidence of sternal trauma increased from 0.7 to 4% (seat-belt syndrome).^[2]^ The motivation for the present case report is based on the paucity of the occurrence of this type of traumatic lesion, the success of a surgical approach (simple and reproducible) and costs of the management of the patient. The aim of this study was to describe a new technical approach to the stabilisation of the MSJ dislocation.

## Case

**Fig. 1:**
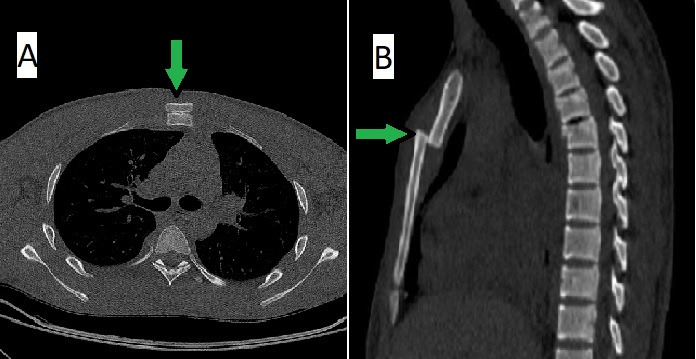
Standard chest X-rays (A) and CT scan (B) confirming the diagnosis of manubriosternal disruption.

**Fig. 2:**
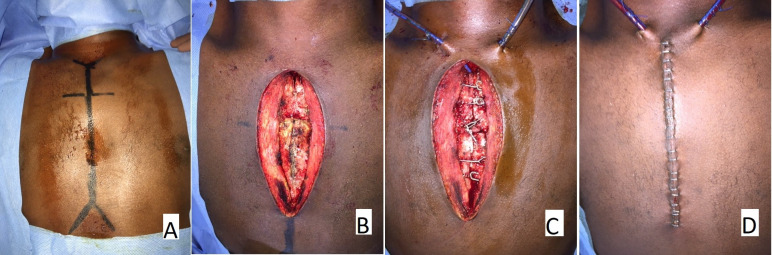
(A) Anatomical landmarks. (B) Perioperative image of manubriosternal dislocation showing incision of the skin, cutaneous layer and periosteum according to landmarks. (C) Sternal closure using stainless steel wires. (D) Postoperative image showing intra-mediastinal drains with six steel wires

**Fig. 3:**
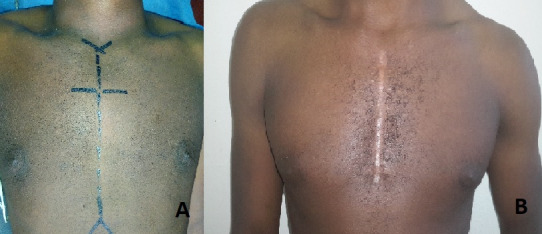
Clinical picture of the patient before surgery and 6 months later

A 19-year-old Comorian man was referred to the Thoracic Surgery Unity at the Joseph Ravoahangy Andrianavalona Hospital after complaining of chest pain at the MSJ that was exacerbated by slight movements. The man had fallen from a mango tree and had his neck flexed a week prior. There was no history of loss of consciousness at the time of injury. The airway, breathing and circulation were intact. During physical examination, he was conscious with stable vital signs. A careful palpation revealed a ‘stair step’-looking deformity located at the anterior chest wall at the level of the MSJ. The examination of the cardiovascular system and electrocardiography were normal. The cardiac contusion markers revealed no pathology. The frontal chest X-ray (CXR) and cervical spine were normal. Standard CXR and computed tomography (CT) scans confirmed the diagnosis of manubriosternal disruption (Fig. 1). It was decided to fix the sternal dislocation under general anaesthesia with single lumen intubation in a supine position (Fig. 2). He made an uneventful recovery. The recovery process involved the removal of the drainage tube, enhancing the mobilisation of the patient and discharge after complete wound healing. To ensure a correct surgical reduction, a CXR was carried out in two planes immediately post operation. The patient was discharged home on day 2 post operation and returned to his work as physiotherapist after 3 weeks. No. complications were observed at 4 weeks and 6 months of follow-up care (Fig. 3).

## Surgical Technique

**Fig. 4:**
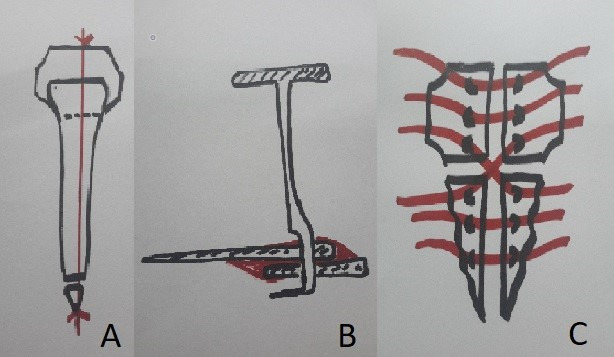
Anatomical landmarks (jugulum and xiphoid). (A) Median sternotomy with Lebsche sternotomie. (B) Sternal closing with six steel wires gauge number 5. (C) MSJ stabilisation with one X-shaped steel wire.

The patient was positioned supine with a chopping block under the tip of the scapula, the arms were placed along the body and general anaesthesia was administered. Prior to the incision, the correct anatomical landmarks (jugulum and xiphoid) were identified (Fig. 4A). We then undertook a median skin incision and a subcutaneous cauterisation from the fossa jugularis to the xiphoidis, followed by a layer-by-layer dissection down to the sternum to reach the MSJ. Once the level of the dislocation was identified, the pectoral muscles were dissected up to the manubrial margins, and the dislocation site was completely exposed. Preceding the osteotomy, the anaesthetist stopped the ventilation to avoid accidental opening of the pleura that can lead to the injury of the underlying mediastinal structure. The osteotomy was performed the whole length of the sternum, just as in the case for a conventional sternotomy (with Lebsche sternotomy) (Fig. 4B). The sternum was extracted progressively, followed by the detachment of the dislocated MSJ, using the periosteal elevator for callus formation until the two fragments were freed up so as to reduce the dislocation anatomically through a median sternotomy access (the removal of the abnormal bone callus). Bleeding was controlled with cautery of periosteum to avoid continuous blood loss during surgery. A chondrectomy was carried out at the joint of the lower edge of the manubrium and the upper edge of the sternum. Six 5-gauge sternal steel wires were placed on each of the sternal edges, either from the top to the bottom of the sternum or in the reverse direction, depending on the surgeon’s preference. We applied one X-shaped steel wire through the manubrium and sternal corporeal, on the level of the manubrio-corporeal joint so that the posterior and anterior side of the joint located at the sternal closure presents an X figure. The other steel wires were placed horizontally through the corpus manubriosternal (Fig. 4C). The sternal steel wires were set tight together on the two fragments of the sternum. The steel wires were tightly pulled together and twisted, burying the cut ends in the soft tissue that is lateral to the sternum. The wound was irrigated with 0.9% saline and two 16 French suction drainage tubes (Blake drain) were placed below the sternum to avoid blood or serous fluid collection. Each anatomical layer was then closed anatomically with resorbable sutures (pectoralis fascia, subcutaneous layer and skin). Particular care was taken to close the pectoralis fascia using 1.0 vicryl suture in a way to form a figure eight that looks interrupted to avoid any dehiscence, soft-tissue erosion and minimising any fascial defects. The subcutaneous tissues were closed with a 2.0 vicryl suture and surgical staples.

## Discussion

An MSJ dislocation may have the form of a sternal dislocation or a sternal segment dislocation. This is an extremely rare traumatic pathology and there is no consensus in the literature on the standard management approach. This poses a challenge in the indications and surgical techniques for stabilisation of this traumatic lesion.^[3]^

Despite the rarity of the lesion and the lack of consensus, the literature is unanimous regarding the operative indications. Compared with the cases of sternal fractures, we found three key points for operative approaches in the literature: (a) the presence of a sternal deformity; (b) loss of sternal continuity for a period >6 weeks; and (c) the persistence of chest pains for 2 - 8 weeks following the trauma.^[4,5]^

Based on our case report and the literature, we suggest classifying these indications according to the diagnostic delay: (i) acute-phase observation is characterised by persistent pain and instability of the anterior chest wall for a period exceeding 7 days, and the presence of associated lesions such as fractures of the costal arches, vertebral spine or blood vessel lesions that may also lead to indications for surgical treatment; and (ii) the late-phase observation (beyond 4 weeks) consisting of chronic non-union, MSJ instability and anterior chest wall deformity.

Our surgical approach obviates some of the surgical technical difficulties and complications that are associated with the use of operative procedures such as osteosynthesis plates and screws, Kirschner or Steinmann wire, the use of bioabsorbable materials or bone grafting and the need for a repeat operation to remove the implants.^[6]^

The potential benefits of surgical stabilisation (osteosynthesis) include reduced mechanical ventilation support, shortened intensive care unit stays and hospitalisation, better secretion management through efficient cough, and minimised chest wall deformities resulting from dislocation. These injuries may lead to septic arthritis,^[8]^ periarticular calcification, ankylosis, chronic pain and structural deformity if left untreated.^[1]^

Our surgical technique for stabilisation of the MSJ has not been described in the literature and presents several advantages, including correction of thoracic deformity and antalgic outcomes, stabilisation of the joint while preserving the dynamics of the thoracic cage, prompt functional recovery of dynamic respiration, and reduction of costs and duration of hospital stay.

The stabilisation of the MSJ dislocation with this open reduction method combined with internal fixation may be achieved by various fixation methods including wire cerclage, suture repair, and plating or alternative rigid fixation devices.^[1]^ Plating may provide enhanced MSJ rigidity while requiring less tissue dissection in comparison with wire fixation.^[1]^

A study by Salloum *et al*.^[13]^ showed that sternal wires are not good and they need to be removed and replaced with metallic plates for fixation 2 months after the initial fixation with wires. Furthermore, K-wire fixation is thought to be risky because of the danger of pin migration as well as breakage of the K-wires.^[14]^ Additionally, K-wire fixation has a mechanical disadvantage because it does not resist flexion and extension of the thorax.^[3]^ Lemaitre *et al*.^[14]^ reported stabilisation of the MSJ with two mattress sutures using heavy polydioxanone ropes (PDS, USA) which are more elastic than wires, less likely to break and also not likely to create an artefact on nuclear magnetic resonance or CT imaging.

In our clinical practice, we used metal wires that result in an easy stabilisation technique that we named 'splitting open-closure of the sternum' technique. This technique resulted in well-healed wounds without postoperative complications and on follow-up assessments. Moreover, the drains could be easily removed.

Patients with sternal fractures and manubriosternal dislocation have been treated with surgical procedures using steel wire and titanium plates for patients with demineralised bone matrix. Divisi *et al*.^[15]^ reported that using titanium plates provided the safe intraoperative and postoperative management compared with steel plates and steel wires.

The technique we developed has low costs. In fact, a previous study reported that the use of steel wires is cheaper that titanium plates with demineralised bone matrix (EUR3 553.60 v. EUR6 047.60) and the incremental cost-effectiveness ratio revealed that titanium plate costs were EUR624.66 more expensive than steel wire per quality-adjusted life-years gained.^[15]^

Our technique achieved stability of the MSJ similar to that obtained by Casha *et al*.,^[16]^ who performed a median sternotomy closure technique. They assessed and quantified the rigidity of sternotomy fixation using a mechanical model and tested six different fixation techniques including figure-of-eight, straight, ethibond, repair, sternaband and multitwist. They also suggested closure of the sternum with a minimum of six straight and four multitwists or four figure-of-eight wires to ensure the stability of the anterior chest wall. Strengthening of the sternal closure by increasing the number of wires also resolved the problem of sternal dehiscence.^[17]^

### Study Limitations

The major limitations of this study are that the surgical approach cannot be generalised, the danger of over-interpretation, publication bias and a retrospective design. Moreover, it is difficult to test our technique in a large clinical trial due to the lack of financial resources and the rarity of patients with these traumatic lesions. Finally, access to median sternotomy and surgical technical platform is a huge problem in developing countries.

## Conclusion

There is no consensus in the literature about the preference of surgical or conservative approaches for the treatment of MSJ dislocation (Table. 1). The conservative treatment is associated with significant rates of recurrent subluxations or luxations, and can lead to chronic pain, periarticular calcification and progressive deformity.^[7]^ However, there are reports demonstrating good outcomes with conservative treatment that is carried out only with observation of the lesion or manipulations to obtain a reduction (Table. 1).^[6,18,19]^ Decisions to pursue surgery should take into account the stability of the joint, the overall health of the patient and associated injuries, patient preference, and the experience of the surgeon.^[3]^ The surgical technique that we developed is indicated in all types of isolated MSJ dislocation, it is easily reproducible and can be adapted to all surgical centres which train in general surgery.

## Figures and Tables

**Table 1:** Management of traumatic manubriosternal dislocation

Years	Age (years), gender	Authors and title of study	Type	Management approach	Outcomes	Removal osteosynthesis
2001	17, male	Smith *et al.*^[6]^ Manubriosternal joint disclocation in contact sport	II	No reduction. Conservative treatment with rehabilitation programme	He has been advised to avoid contact sport in the short term and is being followed-up at regular periods with lateral chest radiographs and clinical examination.	-
2004	50, male	Lemaitre *et al.*^[14]^ Manubriosternal disjunction: A new approach for surgical repair	I	A midline incision two mattress sutures carried out with heavy. polydioxanone ropes.	Satisfactory outcome with well-aligned and stable sternal healing 5 months after operation.	None
2007	15, female	Pidcoe *et al.*^[19]^ Rehabilitation of an elite gymnast with a type II manubriosternal dislocation	II	Conservative treatment with rehabilitation programme.	Rehabilitation process was a success. The gymnast successfully completed the season and qualified for the regional competition.	-
2012	10, male	Soysal *et al.*^[18]^ Management of sternal segment dislocation in a child with closed reduction	II	Conservative treatment with simple extension manoeuvre	Successful management	-
2016	22, male	Wood *et al.*^[3]^ Operative reduction and fixation of a sternomanubrial dislocation: A case report and literature review	II	A longitudinal midline incision was centred over the sternomanubrial joint from the sternal notch towards, but not extending to the xyphoid process	The patient achieved good pain relief and regained physical function	Yes
2017	32, female	Salloum *et al.*^[13]^ Surgical management of traumatic manubriosternal dislocation with locking compression plate	I	Surgical approach with locking compression plate	Pain-free with full range of motion in upper limbs after 6 months	Yes
2019	35, female	Sarkeshik *et al.*^[1]^ Manubriosternal joint dislocation due to blunt force trauma	II	A 5 cm longitudinal incision, sternal fixation plates and screws with two 8-hole Blu SternaLock straight fixation plates	Postoperative pain on visual analogue scale was rated 2/10, which had improved from 9/10 preoperatively. She was discharged on postoperative day 4 and continues to do well 12-month follow-up	Yes
